# Comparison of Durability Between Rezum Water Vapor Therapy and UroLift in Treating Benign Prostatic Hyperplasia: A Multicenter Propensity Score-Matched Analysis

**DOI:** 10.7759/cureus.80914

**Published:** 2025-03-20

**Authors:** Chye-Yang Lim, Chih-Cheng Lai, Ya-Wen Tsai

**Affiliations:** 1 Division of Urology, Department of Surgery, Chi Mei Medical Center, Tainan, TWN; 2 Department of Intensive Care Medicine, Chi Mei Medical Center, Tainan, TWN; 3 School of Medicine, College of Medicine, National Sun Yat-sen University, Kaohsiung, TWN; 4 Division of Preventive Medicine, Chi Mei Medical Center, Tainan, TWN

**Keywords:** benign prostatic hyperplasia, complications, durability, reintervention, rezum water vapor therapy, trinetx database, urolift

## Abstract

Background

Rezum water vapor therapy and UroLift are among the minimally invasive surgical therapies (MISTs) gaining popularity in the treatment of benign prostatic hyperplasia (BPH). This study aims to evaluate and compare the reintervention rates, a measure of durability, for Rezum water vapor therapy and UroLift as MISTs for BPH.

Methods

We conducted a retrospective cohort study using data from the TriNetX Global Collaborative Network, a large database of electronic health records from January 2014 to June 2024. Current Procedural Terminology (CPT) and International Classification of Diseases 10th Revision codes (ICD-10) were used to build the cohorts of men aged over 18 years who underwent either Rezum water vapor therapy or UroLift. Reintervention rates and complication profiles were evaluated over a follow-up period of up to five years.

Results

Cumulative reintervention rates were collected for both Rezum water vapor therapy and UroLift at the 1st, 3rd, and 5th years (2.83% vs. 3.59%, 5.99% vs. 8.76%, 6.81% vs. 10.85%). The average annual increase rate was 1% for Rezum water vapor therapy compared with 1.82% for UroLift, respectively. Most complications were more prominent in the Rezum water vapor therapy group, with urinary retention accounting for 23.42%.

Discussion

Rezum water vapor therapy demonstrates a more durable effect with lower reintervention rates compared to UroLift, based on this large multicenter cohort study. The higher reintervention rate observed with UroLift may reflect differences in the mechanisms of action between the two procedures.

Conclusions

These findings elucidate the superiority of Rezum water vapor therapy in sustaining the therapeutic effect over the long term compared to UroLift. However, more complications were noted in the Rezum water vapor therapy group. Thus, clinicians should take into account the durability and complication profiles in shared decision-making when considering MISTs for BPH.

## Introduction

Benign prostatic hyperplasia (BPH) is the most common benign disease affecting the male genitourinary system, with its prevalence increasing with age [1]. The prevalence of lower urinary tract symptoms (LUTS) ranges from 10% to 30% among men in their 60s and 70s, and rises to approximately 30% in men aged 80 and above [2]. For those with LUTS secondary to BPH, medical treatment is always the first choice, but some patients may require surgical intervention if conservative management fails to provide adequate improvement or significant complications arise [3]. Indications for surgical intervention include severe bothersome symptoms, recurrent urinary tract infections, bladder stones, renal impairment, and other complications.

Traditional surgical treatments for BPH, such as transurethral resection of the prostate (TURP), have proven effective but carry the risk of substantial adverse effects, including urinary incontinence, retrograde ejaculation, and sexual dysfunction [4-5]. Later, laser-based treatments such as vaporization or enucleation emerged; these improved the adverse effects profile compared to traditional TURP but still carried risks of bleeding, TUR syndrome, and others [6]. In recent years, minimally invasive surgical therapies (MISTs) have emerged as alternatives to traditional surgical approaches, offering the potential for reduced morbidity and preservation of sexual function [7]. According to the American Urological Association (AUA) guidelines for the management of LUTS due to BPH, UroLift and Rezum water vapor therapy could be considered for the treatment of prostate sizes between 30-80g in those who desire the preservation of erectile and ejaculatory function [8]. Previous studies have shown that these two types of MISTs brought improvements in the International Prostate Symptom Score (IPSS), maximal flow rate (Qmax), and quality of life (QoL scores) for patients, with relatively low complication rates and minimal impact on sexual function [9].

Rezum water vapor therapy employs convective radiofrequency to generate stored thermal energy in the form of steam. This steam is then delivered transurethrally into the transition zone of the prostate, where it ablates tissue, thereby reducing LUTS [10]. On the other hand, the UroLift procedure uses small implants to lift and hold the enlarged prostate lobes in an open position, thus reducing compression on the urethra and improving urine flow [11]. Some cases could be managed in an office setting under local anesthesia or intravenous sedation, as well as in the form of outpatient surgery.

However, the comparison of the efficacy and durability between the two treatments from a large real-world dataset has not been well-established. Previous studies showed that the comparison results were not conclusive due to the small sample size, short follow-up, or lack of a head-to-head trial. According to a study by Law YX et al., the UroLift procedure had a higher reintervention rate of 8.1% compared to 0% for the Rezum water vapor therapy group within two years [12]. Furthermore, other studies have reported a retreatment rate of up to 5% for the Rezum water vapor therapy group [13]. A meta-analysis involving approximately 2,000 patients revealed a reintervention rate of 6.0% per year for the UroLift procedure, which increased further with longer follow-up durations [14]. Therefore, the long-term durability of these two MISTs remains questionable.

The primary objective of this study was to compare the efficacy and safety of Rezum water vapor therapy and UroLift procedures for the treatment of BPH using data from the TriNetX Research Network, a large real-world electronic health record database. This analysis aimed to provide a comprehensive evaluation of the clinical outcomes and durability of these two minimally invasive surgical interventions in a real-world setting.

## Materials and methods

The data used in this study was collected on December 17, 2024, from the TriNetX Global Collaborative Network, which provided access to electronic medical records from 144 healthcare organizations. TriNetX is a global health research network that links clinical data from healthcare organizations to accelerate clinical research. It includes information on diagnoses, procedures, medications, laboratory results, and other clinical data for patients who were seen at partnered healthcare institutions.

This retrospective study was exempted from Institutional Review Board approval. The data reviewed is a secondary analysis of existing data, does not involve intervention or interaction with human subjects, and is de-identified per the de-identification standard defined in Section §164.514(a) of the HIPAA Privacy Rule. The process by which the data is de-identified is attested to through a formal determination by a qualified expert as defined in Section §164.514(b)(1) of the HIPAA Privacy Rule. This formal determination by a qualified expert was refreshed in December 2020.

**Figure 1:**
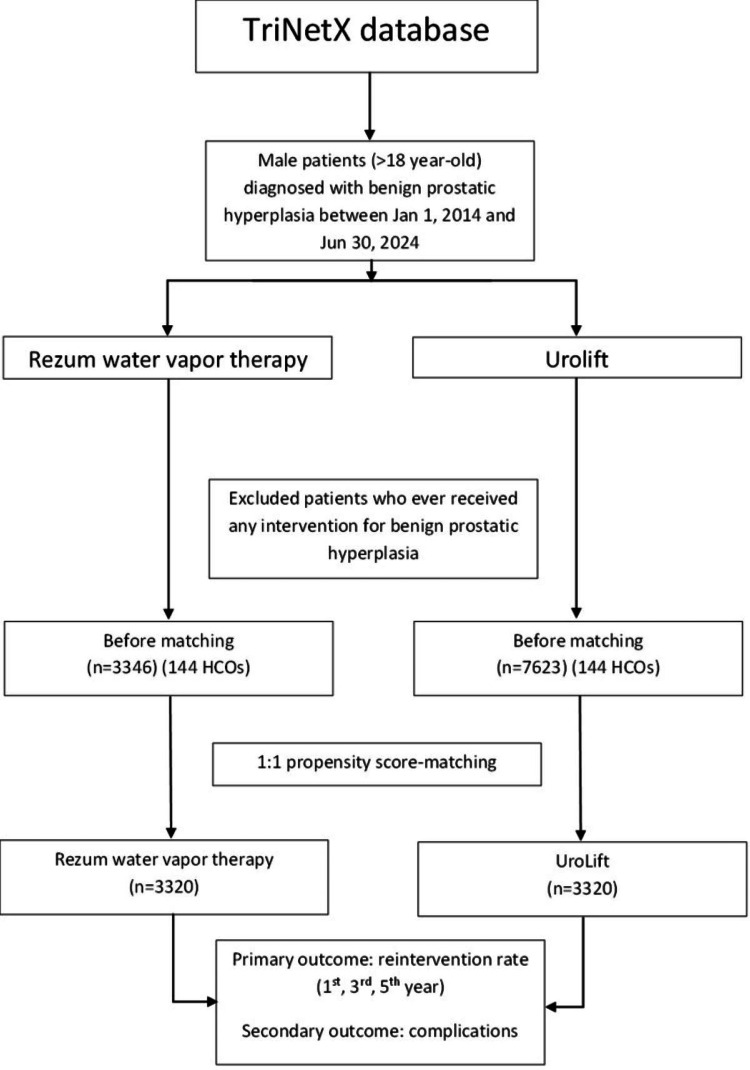
Patient enrollment consort flow diagram.

The study population comprised all adult patients (aged 18 years or older) who underwent either Rezum water vapor therapy or UroLift procedures for the treatment of benign prostatic hyperplasia and were included in the TriNetX research database from January 1, 2014, to June 30, 2024, as shown in Figure 1. We used the International Classification of Diseases, 10th Revision (ICD-10), to identify male patients with benign prostatic hyperplasia (N40). Patients who had received any intervention for benign prostatic hyperplasia prior to the index procedure were excluded. Then we used Current Procedural Terminology (CPT) codes to divide the patients into the Rezum water vapor therapy group (53854)(n=3346) and the UroLift group (52441 and 52442)(n=7623) (Table 1). Meanwhile, demographics of the patients such as age, races, comorbidities, prostate-specific antigen, body mass index (BMI), medications for BPH, anticoagulants, etc., were collected. Then, propensity score matching (PSM) was done on a 1:1 ratio between Rezum and UroLift groups to reduce confounding.

**Table 1:** International Classification of Diseases, Systematized Nomenclature of Medicine, RxNorm, and Current Procedural Terminology Codes.

International Classification of Diseases (ICD) Code	Outcome	
N40	Benign prostatic hyperplasia	
E11	Diabetes Mellitus	
I10	Hypertension	
J40-J47	Chronic lower respiratory disease	
I50	Heart failure	
I25.2	Myocardial infarction	
E65-E68	Overweight, obesity and other hyperalimentation	
R31	Hematuria	
R30.0	Dysuria	
R33	Retention	
R50.9	Fever	
R36.1	Hematospermia	
N53.14	Retrograde ejaculation	
N52	Erectile dysfunction	
N39.0	Urinary tract infection	
SNOMED	Outcome	
4525004	Readmission within 30 days	
RxNorm	Outcome	
720825	Silodosin	
77492	Tamsulosin	
8629	Prazosin	
228790	Dutasteride	
25025	Finasteride	
358263	Tadalafil	
1191	Aspirin	
32968	Clopidogrel	
1037042	Dabigatran etexilate	
1114195	Rivaroxaban	
1364430	Apixaban	
1599538	Edoxaban	
Current Procedural Terminology (CPT) Code	Outcome	
53854 (Rezum Water Vapor Therapy )	Transurethral destruction of prostate tissue; by radiofrequency generated water vapor thermotherapy	
52441 (Urolift)	Cystourethroscopy, with insertion of permanent adjustable transprostatic implant; single implant	
52442 (Urolift)	Cystourethroscopy, with insertion of permanent adjustable transprostatic implant; each additional permanent adjustable transprostatic implant (List separately in addition to code for primary procedure)	
52601 (Transurethral resection of prostate, TURP)	Transurethral electrosurgical resection of prostate, including control of postoperative bleeding, complete (vasectomy, meatotomy, cystourethroscopy, urethral calibration and/or dilation, and internal urethrotomy are included)	
52648 Photoselective vaporization of prostate, PVP)	Laser vaporization of prostate, including control of postoperative bleeding, complete (vasectomy, meatotomy, cystourethroscopy, urethral calibration and/or dilation, internal urethrotomy and transurethral resection of prostate are included if performed)	
52649 (Holmium laser enucleation of the prostate, Holep)	Laser enucleation of the prostate with morcellation, including control of postoperative bleeding, complete (vasectomy, meatotomy, cystourethroscopy, urethral calibration and/or dilation, internal urethrotomy and transurethral resection of prostate are included if performed)	
53852 (Transurethral needle ablation of the prostate, TUNA)	Transurethral destruction of prostate tissue; by radiofrequency thermotherapy	
0421T (Waterjet Ablation)	Transurethral waterjet ablation of prostate, including control of post-operative bleeding, including ultrasound guidance, complete (vasectomy, meatotomy, cystourethroscopy, urethral calibration and/or dilation, and internal urethrotomy are included when performed)	
53850 (Transurethral microwave thermotherapy, TUMT)	Transurethral destruction of prostate tissue; by microwave thermotherapy	
52647 (Interstitial laser coagulation)	Laser coagulation of prostate, including control of postoperative bleeding, complete (vasectomy, meatotomy, cystourethroscopy, urethral calibration and/or dilation, and internal urethrotomy are included if performed)	
52450 (Transurethral incision of the prostate, TUIP)	Transurethral incision of prostate	

The primary outcome is the reintervention rate after the Rezum or UroLift procedure. CPT codes for the procedure of reintervention include Rezum Water Vapor Therapy (53854), UroLift (52441 and 52442), transurethral resection of the prostate (TURP; 52601), photovaporization of the prostate (PVP; 52648), Holmium laser enucleation of the prostate (HoLEP; 52649), transurethral needle ablation of the prostate (TUNA; 53852), transurethral microwave thermotherapy (TUMT; 53850), Waterjet Ablation (0421T), interstitial laser coagulation (52647), and transurethral incision of the prostate (TUIP; 52450). Furthermore, we conducted a subgroup analysis to evaluate the reintervention rates across different age cohorts. The secondary outcome is the complications that may occur following these MISTs. 

## Results

**Figure 2:**
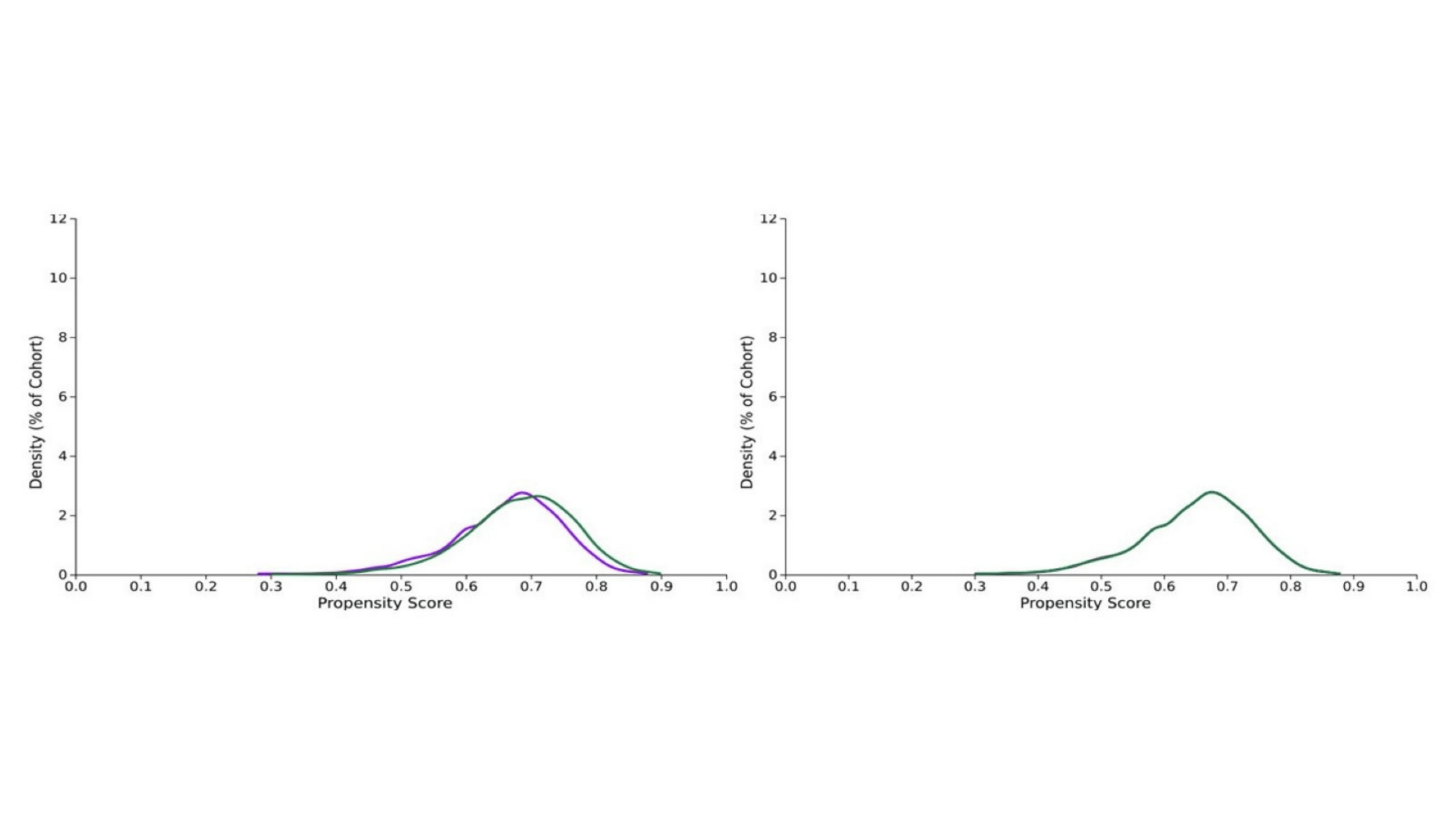
Propensity score density function - before and after matching (Rezum: purple, UroLift: green).

The average age at the time of the first Rezum water vapor therapy and UroLift procedures is 69.4 ± 9.0 (n=3,346) years and 67.5 ± 9.6 years (n=7,623), respectively. After propensity score matching, there were 3,320 patients in each group as shown in Table 2 and Figure 2. Among the patients included, most of them are white men in both groups, with more than 80%, followed by Black or African American and then Asian. More prevalent lower respiratory diseases and obesity were observed in the UroLift group, with statistically significant differences. A higher prostate-specific antigen (PSA) value greater than 4 ng/mL was noted in the UroLift group than in the Rezum water vapor therapy group. Patients in the UroLift group exhibited higher utilization of pharmaceutical therapies for BPH, including medications such as Tamsulosin, Finasteride, Dutasteride, and Tadalafil, compared to the Rezum water vapor therapy group. However, the consumption of antiplatelet or new oral anticoagulants (NOACs) showed no difference.

**Table 2:** Baseline characteristics of patients before and after propensity score matching. NOACs: New oral anticoagulants.

Variables	Before Matching		P-value	SD	After Matching		P-value	SD
	Rezum (n = 3,346)	UroLift (n = 7,623)			Rezum (n =3320)	UroLift (n =3320)		
Age, mean (SD), years	69.4 ± 9.0	67.5 ± 9.6	<0.001	0.201	69.3 ± 9.0	69.3± 9.4	0.853	0.005
Races								
White	2,732 (81.6)	6,097 (80.0)	0.042	0.042	2,711 (81.7)	2,755 (83.0)	0.157	0.035
Black or African American	155 (4.6)	498 (6.5)	<0.001	0.083	155 (4.7)	142 (4.3)	0.44	0.019
Asian	107 (3.2)	191 (2.5)	0.04	0.04	105 (3.2)	90 (2.7)	0.276	0.027
Unknown	241 (7.2)	628 (8.2)	0.064	0.039	228 (6.9)	233 (7.0)	0.598	0.013
Diabetes mellitus	673 (20.1)	1,649 (21.6)	0.073	0.037	667 (20.1)	675 (20.3)	0. 807	0.006
Hypertensive diseases	1,635 (48.9)	3884 (51.0)	0.756	0.042	1,624 (48.9)	1,615 (48.6)	0.825	0.005
Chronic lower respiratory diseases	471 (14.1)	1,301 (17.1)	<0.001	0.083	468 (14.1)	466 (14.0)	0.944	0.002
Heart failure	222 (6.6)	520 (6.8)	0.72	0.007	221 (6.7)	207 (6.2)	0.484	0.017
Obesity	514 (15.4)	1440 (18.9)	<0.001	0.094	513 (15.5)	504 (15.2)	0.759	0.008
BMI, mean (SD), kg/m2	28.4 ± 5.1 (69.8)	29.0 ± 5.4 (60.8)	<0.001	0.115	28.4 ± 5.1 (69.6)	28.4 ± 5.2 (65.4)	0.486	0.021
Prostate specific antigen (SD), ng/mL	3.1 ± 10.7 (55.0)	2.7 ± 10.6 (50.8)	0.181	0.038	3.1 ± 10.8 (54.8)	3.2 ± 14.7 (55.3)	0.819	0.008
≤4 ng/mL	1,545 (46.2)	3,424 (44.9)	0.223	0.025	1,531 (46.1)	1,568 (47.2)	0.363	0.022
>4 ng/mL	510 (15.2)	784 (10.3)	<0.001	0.149	495 (14.9)	491 (14.8)	0.89	0.003
Medications for lower urinary tract symptoms								
Alfuzosin	372 (11.1)	787 (10.3)	0.213	0.026	366 (11.0)	368 (11.1)	0.938	0.002
Terazosin	101 (3.0)	180 (2.4)	0.045	0.041	98 (3.0)	96 (2.9)	0.884	0.004
Doxazosin	87 (2.6)	256 (3.4)	0.036	0.045	86 (2.6)	84 (2.5)	0.877	0.004
Silodosin	164 (4.9)	346 (4.5)	0.406	0.017	163 (4.9)	157 (4.7)	0.731	0.008
Tamsulosin	1,925 (57.5)	4,715 (61.9)	<0.001	0.088	1,912 (57.6)	1,860 (56.0)	0.198	0.032
Finasteride	928 (27.7)	1,724 (22.6)	<0.001	0.118	910 (27.4)	869 (26.2)	0.256	0.028
Dutasteride	171 (5.1)	253 (3.3)	<0.001	0.089	155 (4.7)	144 (4.3)	0.515	0.016
Tadalafil	438 (13.1)	1,253 (16.4)	<0.001	0.094	437 (13.2)	448 (13.5)	0.691	0.01
Antiplatelet or NOACs								
Aspirin	916 (27.4)	2,044 (26.8)	0.541	0.013	911 (27.4)	871 (26.2)	0.268	0.027
Clopidogrel	220 (6.6)	501 (6.6)	0.996	<0.001	219 (6.6)	204 (6.1)	0.451	0.019
Rivaroxaban	99 (3.0)	209 (2.7)	0.526	0.013	98 (3.0)	98 (3.0)	1	<0.001
Apixaban	225 (6.7)	436 (5.7)	0.042	0.042	221 (6.7)	208 (6.3)	0.516	0.016
Edoxaban	10 (0.3)	10 (0.1)	0.058	0.036	10 (0.3)	10 (0.3)	1	<0.001

**Figure 3:**
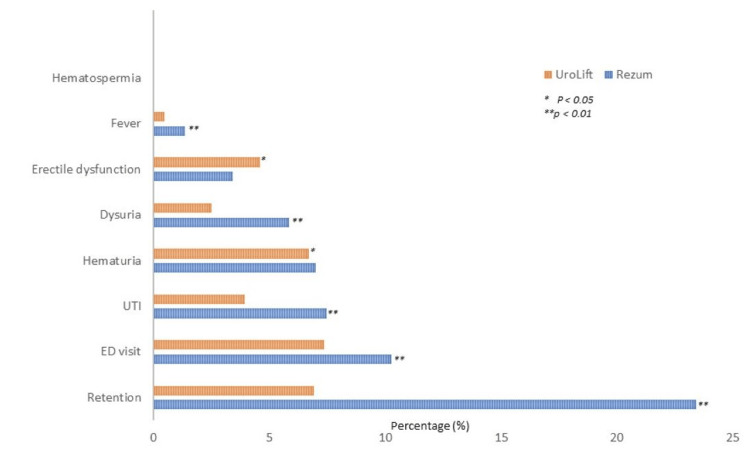
Complications of patients.

Patients in the Rezum water vapor group had higher rates of hematuria, dysuria, urinary retention, fever, erectile dysfunction, urinary tract infections, and emergency department visits compared to the UroLift group following the procedures, as shown in Table 3 and Figure 3. Hematospermia was not observed in either group. Urinary retention was the most common complication, with an incidence of 23.42% in the Rezum water vapor group. Meanwhile, fever episodes were much more frequent in the Rezum water vapor group, with the HR being 2.99 compared to the UroLift group.

**Table 3:** Complications of patients after propensity score matching. χ²: Chi-square; df: Degrees of freedom.

Complications	Rezum	UroLift	Hazard Ratio (95% CI)	χ^2^	df	P-value
Hematuria	230 (6.97)	171 (6.7)	1.33 (1.09, 1.67)	5.331	1	0.0049
Dysuria	193 (5.85)	111 (2.5)	1.73 (1.37, 2.19)	21.540	1	<0.0001
Retention	773 (23.42)	525 (6.9)	1.46 (1.31, 1.64)	47.740	1	<0.0001
Fever	45 (1.36)	15 (0.45)	2.99 (1.67, 5.37)	14.921	1	0.0001
Hematospermia	0	0	-	-	-	-
Erectile dysfunction	112 (3.39)	151 (4.57)	0.73 (0.57, 0.94)	4.706	1	0.0126
UTI	246 (7.45)	129 (3.91)	1.91 (1.55, 2.37)	56.441	1	<0.0001
90 days ED visit	338 (10.24)	242 (7.33)	1.39 (1.18, 1.64)	11.069	1	<0.0001

**Figure 4:**
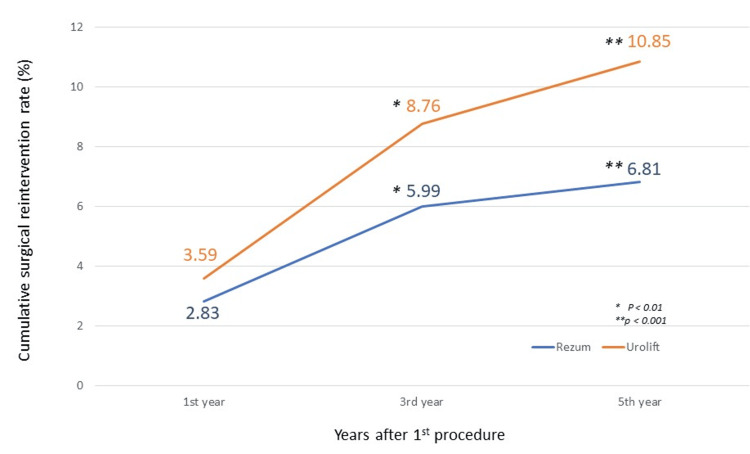
Cumulative surgical reintervention rate within 5 years.

Regardless of age, the reintervention rate for Rezum water vapor therapy showed 2.83% at 1st year, 5.99% at 3rd year, and 6.81% at 5th year. While the reintervention rate for the UroLift group was 3.59% at 1st year, 8.76% at 3rd year, and 10.85% at 5th year, as shown in Figure 4. Although the reintervention rate of UroLift was higher than Rezum during the 1st year, the reintervention rate for both groups did not differ significantly. However, at the 3rd and 5th year, the reintervention rate for UroLift was statistically higher than that for the Rezum water vapor therapy group (Table 4).

**Table 4:** Subgroup analysis of cumulative surgical reintervention rate by age within five years.

Age	Years after 1st intervention	Rezum	UroLift	Hazard Ratio (95% CI)	P-value
	1st	94 (2.83)	119 (3.59)	0.83 (0.64, 1.09)	0.1816
All age	3rd	195 (5.99)	285 (8.76)	0.77 (0.64, 0.92)	0.0049
	5th	226 (6.81)	360 (10.85)	0.74 (0.62, 0,87)	0.0003
	1st	0	0	-	-
50-59 year old	3rd	14 (6.67)	13 (6.19)	1.11 (0.52, 2.37)	0.7799
	5th	14 (6.67)	14 (6.67)	1.05 (0.50, 2.20)	0.9027
	1st	26 (2.67)	25 (2.57)	1.09 (0.63, 1.89)	0.7576
60-69 year old	3rd	57 (6.20)	84 (9.13)	0.76 (0.54, 1.07)	0.11
	5th	73 (7.49)	96 (9.86)	0.84 (0.62, 1.14)	0.2619
	1st	44 (3.33)	46 (3.48)	0.99 (0.66, 1.50)	0.9757
70-79 year old	3rd	77 (5.83)	118 (8.93)	0.70 (0.53, 0.94)	0.015
	5th	89 (6.74)	142 (10.75)	0.70 (0.53, 0.91)	0.0072
	1st	18 (2.47)	24 (3.29)	0.76 (0.41, 1.40)	0.3759
≥80 year old	3rd	39 (5.35)	55 (7.54)	0.74 (0.49, 1.12)	0.1489
	5th	48 (6.58)	67 (9.19)	0.78 (0.54, 1.13)	0.1926

By stratification of age group, only patients between 70-79 years old showed a significant difference in reintervention rates at the 3rd and 5th year between the two groups. UroLift demonstrated a higher reintervention rate of 8.93% versus 5.83% at the 3rd year and 10.75% versus 6.74% at the 5th year compared to the Rezum water vapor group, respectively.

## Discussion

In recent years, the surgical treatment for BPH has increasingly shifted from the conventional transurethral resection of the prostate to the newer MISTs due to being less invasive, offering faster recovery, not requiring general anesthesia, reducing the need for hospitalization, preserving ejaculatory function, and resulting in fewer complications in continence. Both Rezum water vapor and UroLift are comparable MISTs for the treatment of BPH that meet the advantages mentioned. This real-world analysis leveraged a large electronic health record database to provide important insights into the comparative effectiveness and durability of these two procedures. This is the first study comprised of a large number of cohorts, with more than 3,000 patients in each group after propensity score matching, providing a robust sample size to comprehensively evaluate and compare the long-term clinical outcomes and reintervention rates of Rezum water vapor therapy and UroLift in a real-world setting.

Our study found that the cumulative reintervention rate after one year for Rezum is 2.83%. It then increased at an average of 0.99% per year until reaching 6.81% in the fifth year. The reintervention rate for Rezum water vapor therapy demonstrated a statistically significant increase from the first to the third year following the initial procedure by +3.16%. However, the increase slowed thereafter, rising by only an additional 0.82% from year 3 to year 5. This phenomenon is probably due to the maturation of the surgeons' technical skills over time and improvements in patient selection. Initially, surgeons may need more cases to master the techniques and proper patient selection, but after gaining more experience, the reintervention rate becomes more stable. Comparatively, this finding is lower than a systematic review and meta-analysis conducted by Miller LE et al., which showed the cumulative surgical retreatment rate following Rezum water vapor therapy was 7.0% at four-year follow-up [10]. Conversely, another randomized controlled study reported a surgical retreatment rate of 4.4% over four years, which is lower than our study [15]. Additionally, a systematic review and meta-analysis of 15 studies involving 471 men undergoing Rezum therapy for prostates of at least 80 cm^3^ in volume demonstrated excellent long-term durability, with a surgical retreatment rate of only 1.2% [16].

In contrast, the UroLift procedure exhibited a cumulative reintervention rate of 3.59% within the first year after the initial procedure. The cumulative reintervention rate then increased significantly, rising to 8.76% by the third year, a substantial increase of 5.17% over the first-year rate. Moreover, the cumulative reintervention rate for UroLift further escalated to 10.85% at the fifth year. Overall, the reintervention rate of UroLift was always higher than that of the Rezum water vapor group after 1 year of the procedure but only reached statistical significance since the 3rd-year follow-up. Our findings indicate a lower reintervention rate for UroLift compared to a previous study that reported a 17.18% rate of procedure failure requiring further interventions for BPH within two years post-operatively [17]. According to the L.I.F.T. study (five-year results of a prospective, multi-center, randomized, blinded sham control trial of UroLift), the surgical retreatment was 13.6% over 5 years [18]. A systematic review and meta-analysis published in 2024 by Brian Ng Hung Shin et al. also showed that the crude reintervention rate for UroLift in a one-year follow-up was 0-15.8% in four studies reviewed, which is much higher than our study [19]. Another study which utilized the TriNetX database as we did, showed the cumulative reprocedure rates of UroLift after one year was 5.1% (n = 14,343) and 16.1% after four years, with an average annual increase of 3.6% per year [20]. Regarding the age-dependent issue, our study showed there is no significant difference in reintervention rates between Rezum water vapor therapy and UroLift in the early age group (<70 years old) and ≥80 years old. However, for the 70-79-year-old age group, a significantly higher reintervention rate was observed in the UroLift group compared to the Rezum group at both three-year (8.93% vs 5.83%) and 5-year (10.75% vs 6.74%) follow-ups as shown in Table 4.

Previously, there were not many publications directly head-to-head comparing the efficacy and durability between MISTs such as Rezum water vapor therapy and UroLift. Although some studies conducted in recent years, the study populations were relatively small. In this study with a large population included, Rezum water vapor therapy is better than UroLift in terms of the overall reintervention rate from the 1st year, 3rd year, and 5th year (2.83% vs 3.59%, 5.99% vs 8.76%, 6.81% vs 10.85%). Three small cohort studies with short duration follow-ups revealed Rezum water vapor therapy to be superior to UroLift in the context of reintervention (0-2.5% vs 7.35-16%) [21-22].

Several factors contribute to the higher reintervention rate of UroLift compared to Rezum. First, in terms of mechanism of action, UroLift physically lifts and pushes aside the obstructing prostate tissue using small implants [23]. Over time, the prostate can continue to grow, potentially dislodging the implants or rendering them less effective. Rezum, on the other hand, uses thermal energy to ablate prostate tissue, offering a more permanent reduction in obstructing tissue. Secondly, while UroLift effectively opens the prostatic urethra, it doesn't address the underlying issue of prostate enlargement. If a significant portion of the prostate remains enlarged, it can continue to obstruct urine flow, leading to recurrent symptoms and the need for reintervention. Rezum's tissue ablation addresses the size of the prostate more directly, even with the protruding median lobe, potentially leading to better long-term outcomes. Thirdly, although UroLift implants are generally safe, complications such as implant migration, encrustation, or discomfort can occur, potentially requiring reintervention [24]. Lastly, proficiency with the UroLift procedure takes time and experience to develop, as it requires specialized skills and techniques to achieve optimal outcomes. Based on our research, the reintervention rate for UroLift from the 1st year to the 3rd year was 5.17%, but the rate dropped to 2.09% until the 5th year follow-up. Rezum water vapor therapy may have a shorter learning curve compared to UroLift, leading to better overall outcomes.

The rates of adverse events such as 90 days ED visits, hematuria, dysuria, urinary retention, fever, and urinary tract infections were higher in the Rezum water vapor group compared to UroLift as shown in Table 3. However, a previous meta-analysis showed no significant differences between the two procedures in terms of functional outcomes such as IPSS, Qmax, and QoL at 12 months [25]. The higher complication rates observed with Rezum water vapor therapy may be partly attributable to the tissue-ablation mechanism of action, which can lead to a more inflammatory response compared to the tissue-displacing approach of UroLift.

Most of the complications of Rezum water vapor therapy is urinary retention (23.42% vs 6.90%), HR 1.46 (1.31, 1.64) (p<0.0001) [26]. Our result is unconcordant with a previous study which showed over 10% of men undergoing Rezum water vapor therapy for LUTS/BPH will experience trial without catheter (TWOC) failure and acute urinary retention (AUR) after 7 days of catheterization [26]. Mathieu Coscarella et al. found that a preoperative post-void residual (PVR) greater than 120ml is associated with a higher risk of urinary retention after Rezum water vapor therapy, which may explain the higher rate of retention in our study as well. In contrast, the cause of retention in UroLift may be due to blood clot obstruction as previously described in another study [27].

Meanwhile, the rate of UTI in the Rezum water vapor therapy group is also higher than in the UroLift group (7.45% vs 3.91%), which is partly due to a higher number of days of postoperative catheterization. ED visits within 90 days are also high for both Rezum water vapor therapy and UroLift (10.24% vs 7.33%). Although the incidence of erectile dysfunction (ED) was lower in MISTs, our study found that Rezum had an even lower rate of ED compared to UroLift (3.39% vs 4.57%). Previous research has shown no significant differences in sexual function outcomes between patients undergoing Rezum water vapor therapy and UroLift procedures [22].

Despite the higher complication rates, the long-term durability of Rezum water vapor therapy appears superior to UroLift, with lower reintervention rates out to 5 years. This finding aligns with results reported in prior studies, which have consistently demonstrated higher retreatment rates for the UroLift procedure compared to Rezum water vapor therapy over longer follow-up periods [7, 28]. The differing durability between the two procedures may be explained by their distinct mechanisms of action - the Rezum water vapor therapy approach relies on thermal ablation to reduce prostatic tissue, whereas UroLift uses permanent implants to physically displace obstructing prostatic lobes. This comprehensive real-world analysis provides valuable comparative insights that can inform clinical decision-making and guide treatment selection for patients with BPH.

This study has certain limitations that should be acknowledged. Using a database like TriNetX introduces limitations inherent to retrospective studies. Data quality relies on the accuracy and completeness of existing records, which may vary. Selection bias can also occur due to the criteria used to include patients in the database. Furthermore, some patient characteristics or clinical details may not have been fully captured, such as prostate size, IPSS, results of uroflowmetry, and quality of life questionnaires. The details of the surgical procedure, like the number of implants deployed in UroLift or the number of ablations performed in Rezum water vapor therapy, were also not available. These procedure-specific factors could influence outcomes. In addition, the indications and timing for reinterventions were not standardized, and the specific reasons for these follow-up procedures were not always clearly documented.

## Conclusions

The cumulative reintervention rate for Rezum water vapor therapy was lower than for UroLift over a five-year period, indicating better long-term durability. However, Rezum water vapor therapy was associated with a higher rate of certain postoperative complications, including urinary retention and urinary tract infections. Overall, both Rezum water vapor therapy and UroLift offer effective and minimally invasive options for the management of BPH, each with unique trade-offs that clinicians should consider when selecting the optimal treatment approach for their patients. The reintervention rate could also be considered a factor in decision-making and incorporated into future guideline recommendations.
